# Comparison of Age-Stratified Seroprevalence of Antibodies against Norovirus GII in India and the United Kingdom

**DOI:** 10.1371/journal.pone.0056239

**Published:** 2013-02-21

**Authors:** Vipin Kumar Menon, Santosh George, Farah Aladin, Sameena Nawaz, Rajiv Sarkar, Ben Lopman, James J. Gray, Miren Iturriza Gomara, Gagandeep Kang

**Affiliations:** 1 Department of Gastrointestinal Sciences, Christian Medical College, Vellore, India; 2 Virus Reference Department, Centre for Infection, Health Protection Agency, London, United Kingdom; 3 Division of Viral Diseases, National Center for Immunization and Respiratory Diseases, Centers for Disease Control and Prevention, Atlanta, Georgia, United States of America; 4 Norfolk and Norwich University Hospital Specialist Virology Centre, Microbiology Department, NRP Innovation Centre, Norwich, United Kingdom; 5 Department of Clinical Infection, Microbiology and Immunology, Institute of Infection and Global Health, University of Liverpool, Liverpool, United Kingdom; University of Ottawa, Canada

## Abstract

Noroviruses are a common cause of gastroenteritis worldwide, but outbreaks appear to be more common in industrialized countries than in developing countries, possibly reflecting differences in exposure and immunity. In this study, age-stratified sera from India and UK populations were analysed for the presence of norovirus-genogroup II specific IgG by a time resolved immunofluorescence assay and relative levels of antibodies in the two populations were compared. Antibody levels were higher among all age groups in India than in UK and increased with age in India, whereas in the UK, levels of antibody decreased in adulthood. These results indicate different patterns of exposure to noroviruses in the two countries.

## Introduction

Noroviruses (NoV) are a major cause of sporadic gastroenteritis worldwide, as well as of outbreaks in hospitals and semi-closed communities such as residential and nursing homes [1]–[2]. Transmission is mainly via the fecal-oral route, through person-to-person contact or through contaminated food, water or the environment [3]. Infection elicits incomplete and short lived immunity, and previous studies with volunteers demonstrated that although pre-existing serum antibodies to NoV do not protect from re-infection, antibody levels to NoV are associated with protection after repeated exposure [4]. Seroprevalence studies have demonstrated that NoV infections are ubiquitous and infections occur early in life with prevalence reaching >95% in adults [5]–[7].

Reports of NoV prevalence in India are primarily from hospital and community based studies of sporadic acute gastroenteritis [8]–[11] and only one outbreak in a semi-closed setting has been reported [12].

This study examined the levels of human NoV-specific antibodies in age-stratified sera from India and UK populations as a measure of exposure to NoV infections in the two populations throughout life and demonstrated a difference which may, at least in part, explain the low incidence of NoV outbreaks in India in comparison to the UK.

## Materials and Methods

### Ethics Statement

The study was approved by the Institutional Review Boards of the Christian Medical College, Vellore, India. Prior to enrollment, written informed consent was obtained from the parents or legal guardians of eligible children.

### Study Area and Population

In India, 1044 sera included sera collected in a probability proportional to size cluster survey conducted in Vellore from August 1999 to February 2000 [13] (samples from 0–40 years), and sera from the Biochemistry laboratory and the Community Health and Development Hospital, Vellore from residents in geographic areas of the serosurvey (sera from >40 years of age). The studies were approved by the Institutional Review Board of the Christian Medical College, Vellore. In the UK, 1034 sera collected in 2000 or 2005 from the HPA Seroepidemiology Programme (http://www.hpa.org.uk/webw/HPAweb&Page&HPAwebAutoListName/Page/1158313434390?p=1158313434390) were used. Sera from both cohorts were distributed in 5 age groups (Table 1).

**Table 1:** Summary of sera tested.

Age Group	India Cohort	UK Cohort
<6 months	83	47
7 m–5 y	170	160
10–15 y	237	207
20–30 y	189	207
40–45 y	158	206
50–60 y	207	207
Total	1044	1034

### Time Resolved Fluorescence Immunoassay (TRFIA)

Detection of human NoV-specific serum IgG was done by Time Resolved Fluorescence Immunoassay (TRFIA), which incorporates lanthanide labels that confer high sensitivity and a wider dynamic range than ELISA assays [14], and hence allows quantitation without the need for inclusion of a dilution series. Human NoV virus like particles (VLPs) were produced from an epidemic strain GII.4v2 [15], circulating in the UK during 2002 and later detected worldwide, the viral protein 1 (VP1) has 94% amino acid homology to the predominant Indian strain. The VLPs were used at a concentration of 2 µg/ml in 0.05 M carbonate/bicarbonate buffer, pH 9.6 to coat 96-well high-binding microwell plates (Costar, Corning, N.Y.), overnight at 4°C. Plates were washed 5 times with DELFIA wash buffer (PerkinElmer, UK) and 100 µl of serum diluted 1∶100 in DELFIA assay buffer (PerkinElmer, UK) was loaded per test well. Each plate included a standard curve of 8 serial two-fold dilution of a positive serum sample starting at 1∶100, a blank (no serum) and positive control and a negative control (goat serum). The plates were incubated in a humid chamber for 2 h at 37°C and then washed 5 times. A total of 100 µl of europium (Eu) labelled anti-human IgG conjugate (PerkinElmer, UK) diluted 1∶500 in DELFIA buffer was added to each well using a multi-channel pipette, followed by incubation for 1 h at 37°C and washing as before. A total of 150 µl of DELFIA enhancement solution (PerkinElmer, UK) was added to each well and plates were incubated in the dark at room temperature for 15 min with gentle rotation. Plates were read using a DELFIA 1234 reader (PerkinElmer, UK) and data analyzed using Multicalc software, version 4.0 (Wallac Oy, Finland). The distribution of the Eu counts was normalized by performing a natural log transformation. The plate-to-plate variability between the assays was eliminated by standardizing the slope and intercept across all experimental runs of the log-transformed europium counts, against a reference plate. Data were further analysed using STATA 10.0 for Windows (STATA Corp., TX, USA), using student’s t-test and ANOVA, as appropriate.

## Results and Discussion

Using a cut-off of +3SD above the mean of negative controls, the prevalence of human NoV-specific antibody in both cohorts was high, 99.5% and 99.4% in the India and the UK cohort, respectively. With a higher arbitrary Eu count cut-off of 7000, 4% of samples from UK and 1.6% samples from India were below this cut-off, mainly in the 0–5 year age group, with younger children within the group having fewer antibodies. The high prevalence found in this study, particularly for the UK cohort, when compared to previous studies may be due to the use of a more sensitive assay and also the choice of antigen, which used the recently widely circulating GII.4 strain, although a subset of sera were evaluated with a GII.4 VLP made with a pre-2002 strain also showed complete cross-reactivity (data not shown). Other studies have shown that the antibodies detected in human NoV-specific EIA are largely cross-reactive across different NoV genotypes [16]–[19]. Studies examining seroprevalence to GII NoVs reported overall seropositivity of 91.2%, 74.1% 87% and 96.5% in Italy, France, Japan and South Africa, respectively [20]–[23] using conventional ELISAs. A study from China showed that the seropositivity increased from 70% among one year olds to 98% among 8–9 year old children for human norovirus GII and from 65% in the one year olds to 100% among the 8–9 year old children for human norovirus GI [24].

**Figure 1:**
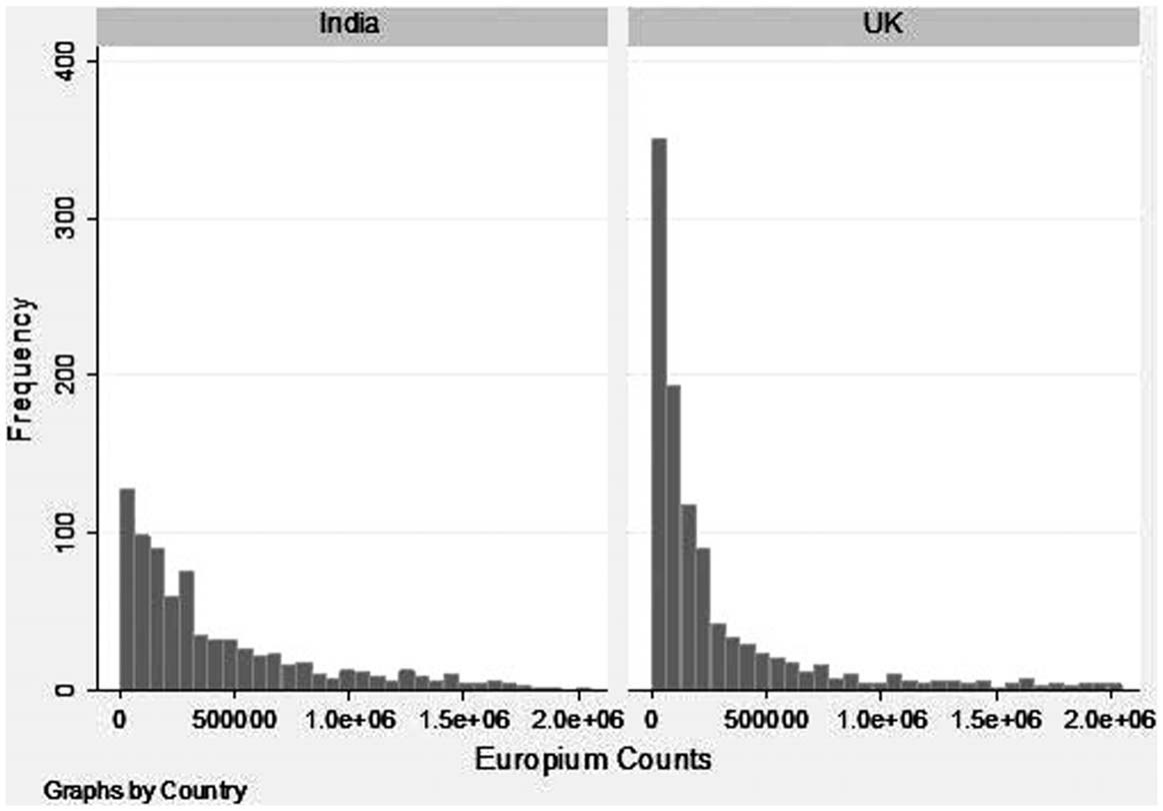
Distribution of Europium counts obtained with sera from the India and UK sera cohorts. The europium counts were log transformed for a normal distribution. The first plate from India was the reference category and a slope and intercept was calculated for all other plates from both India and the UK. The slope and the intercept values were used to standardize the log-transformed europium counts across experimental runs. Standardized log-transformed values were then used for all analyses.

Although the proportion of seropositives to human NoV antibody was comparable in the two populations, the levels of antibodies as represented by the distribution of Eu counts were markedly different (Figure 1). Due to the lack of a standard serum for quatification of human NoV-specific antibodies, the results are presented as relative quantitation of antibody levels between the two cohorts rather than absolute quantification.

**Figure 2:**
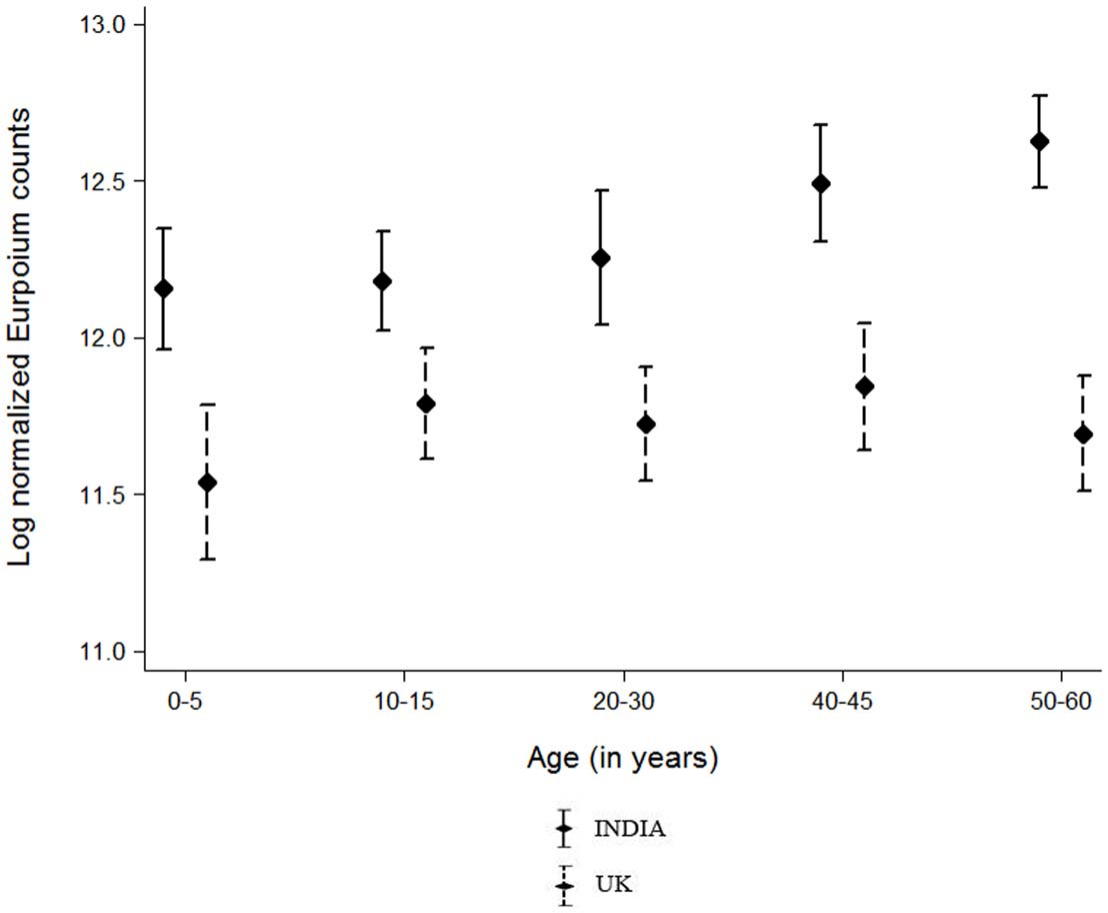
Distribution of log normalized Europium counts among the different age strata in India and the UK. The dots in the center represent the mean Europium counts for that age group whereas the horizontal error bars represent +2 standard deviations. Europium counts were measured by time resolved immunofluresecence assay.

For the comparative analysis between the two cohorts, exclusion of the negative samples did not alter the results, and data shown includes the complete cohorts. Sera from India showed significantly higher levels of norovirus-specific IgG than the sera from UK across all 5 age groups (Fig. 2). The Indian panel showed a steady rise in human NoV-specific antibody levels with age, reaching the highest levels in the age group of 50–60 years of age (Fig. 2). In the UK panel, the antibody levels was lowest among the 0–5 year old children with a mean (SD) log normalized Eu count of 11.5 (1.8), increasing to 11.8 (1.3) in 10–15 years age group; the differences were not statistically significant (P = 0.264). Among the Indian samples, antibody levels were significantly different across age groups, with higher levels among the 50–60 year age group [Mean (SD) 12.6 (1.1)] in comparison to the younger age groups 0–5 [Mean (SD) 12.2(1.5), P = 0.003] and 10–15 year age groups [Mean (SD) 12.2(1.5), P<0.001]. No significant difference was observed (at p = 0.003 level, after Bonferroni correction) for any of the other age groups (Fig. 2). The antibody levels were comparable between males and females for all age groups except the 20–30 year olds. In this age group the antibody levels were higher in females (12.1, 1.4) than in males (11.8, 1.4, *P* = 0.016). Although not statistically significant, this relationship held true for both India [12.4 (1.5) vs. 12.0 (1.5), *P* = 0.102] and UK [11.8 (1.3) vs. 11.6 (1.3), *P* = 0.188] panels (data not shown).

The results show higher levels of human NoV-specific antibodies in the population in India compared to the UK, with the widest difference between the two populations in the youngest and the oldest age groups analysed. These findings suggest more constant exposure to noroviruses across all age groups in India when compared to the UK. A number of factors are known to play a role in the increased prevalence of some infectious diseases in developing countries. These include poor hygiene and sanitation, less access to healthcare, overcrowding as well as socio-cultural differences wherein people in India live in larger family units when compared to developed countries [25]–[28]. Norovirus infections, similar to rotavirus infections, are endemic in infants and children. Recently it has been demonstrated that in India, first rotavirus infections occur earlier than in the middle and high-income countries, and that number of reinfections is higher [29] and that seroprevalence of rotavirus group A antibodies is 100% in the same age-stratified population as evaluated here [30]. The same pattern could be expected for human norovirus infections, which would be consistent with the data presented here and with other studies from developing countries [23]–[24], [31]. In the UK, the peak of human norovirus-specific antibody levels in the 5–10 year olds is likely to reflect increased opportunities for infection through contact with other children. Indeed, in Europe, social contact rates are highest in school age children [32]. The lower human norovirus–specific antibody levels in the >50 years old could also correlate with the increased susceptibility in this age group that translates into the high frequency of norovirus outbreaks that are seen each winter in the UK, particularly among elderly patients in hospitals and residential care institutions. In addition to the social contact across all ages, it is likely that in India there are also greater opportunities for food, water and environmental transmission of noroviruses, contributing to greater and steady exposure and boosting of immunity. This may result in re-infections being mostly subclinical, and outbreaks being rare in this population in contrast to the high incidence of outbreaks involving adults and the elderly in the UK and other high income countries.
